# Supplementary material for: Modelling Skylarks (Alauda arvensis) to Predict Impacts of Changes in Land Management and Policy: Development and Testing of an Agent-Based Model
Source: PLoS One. 2013 Jun 6;8(6):e65803. doi: 10.1371/journal.pone.0065803 (PMC3675089; doi:10.1371/journal.pone.0065803)
Supplement: Supporting Information S4 — The skylark ODdox as a zipped archive. (ZIP) [file pone.0065803.s004.zip › Skylark_ODdox/functions_0x63.html]

ALMaSS Skylark ODdox: Class Members


ALMaSS Skylark ODdox
 2.0


- Main Page
- Related Pages
- Classes
- Files

- Class List
- Class Index
- Class Hierarchy
- Class Members

- All
- Functions
- Variables
- Typedefs

- a
- b
- c
- d
- e
- f
- g
- h
- i
- j
- k
- l
- m
- n
- o
- p
- r
- s
- t
- u
- v
- w
- x
- y
- z
- ~

Here is a list of all class members with links to the classes they belong to:

### - c -

- CalcFoodTime()
  : Skylark\_Female
- CalculateCentroids()
  : Landscape
- CalculateEggNumber()
  : Skylark\_Female
- Calendar()
  : Calendar
- CanGrow()
  : PolygonDataVector
- Catastrophe()
  : Skylark\_Population\_Manager
  , Population\_Manager
- CattleIsOut()
  : Farm
- CattleIsOutLow()
  : Farm
- CattleOut()
  : Farm
- CattleOutLowGrazing()
  : Farm
- CentroidSpiralOut()
  : Landscape
- CfgBase()
  : CfgBase
- CfgBool()
  : CfgBool
- CfgFloat()
  : CfgFloat
- CfgI
  : Configurator
- CfgInt()
  : CfgInt
- CfgStr()
  : CfgStr
- CfgVals
  : Configurator
- ChangeMapMapping()
  : Landscape
- CheckForFields()
  : Skylark\_Female
- CheckManagement()
  : TAnimal
- CheckManagementXY()
  : TAnimal
- CheckRotationManagementLoop()
  : Farm
- CheckXY()
  : Population\_Manager
- CIPEGridOutputPrb
  : Population\_Manager
- CIPEGridOutputPrbB
  : Population\_Manager
- CIPELandscapeMaker()
  : Landscape
- ClaimGrid()
  : SkTerritories
- ClearMap()
  : BinaryMapBase
- ClearMapValue()
  : MovementMap16
  , IDMap
  , SimplePositionMap
  , ScalablePositionMap
  , PositionMap
- ClearValue()
  : BinaryMapBase
- CloseFile()
  : probe\_data
- CloseTheCIPEGridOutputProbe()
  : Population\_Manager
- CloseTheMonthlyRipleysOutputProbe()
  : Population\_Manager
- CloseTheReallyBigOutputProbe()
  : Population\_Manager
- CloseTheRipleysOutputProbe()
  : Population\_Manager
- Clutch\_Size
  : Skylark\_Clutch
- Coast()
  : Coast
- Configurator()
  : Configurator
- ConiferousForest()
  : ConiferousForest
- ConnectFarm()
  : FarmManager
- ConstructAHabitatTable()
  : Skylark\_Male
- ConventionalCattle()
  : ConventionalCattle
- ConventionalPig()
  : ConventionalPig
- ConventionalPlant()
  : ConventionalPlant
- ConvMarginalJord()
  : ConvMarginalJord
- Copse()
  : Copse
- CopyMyself()
  : Skylark\_Adult
  , TAnimal
- CorrectCoords()
  : Landscape
- CorrectHeight()
  : Landscape
- CorrectWidth()
  : Landscape
- CorrectWrapRound()
  : TAnimal
- CountMapSquares()
  : Landscape
- Counts
  : Population\_Manager
- CreateFarms()
  : FarmManager
- CreateObjects()
  : Skylark\_Population\_Manager
- Crop()
  : Crop
- CropData()
  : CropData
- CropGrowth()
  : CropGrowth
- CropNum
  : Starter
  , Rotation
- CropRotation()
  : CropRotation
- CurrentStateNo
  : TALMaSSObject
- Cutting()
  : OrchardGrass
  , Orchard
  , RoadsideVerge
- CutToHay()
  : Farm
- CutToSilage()
  : Farm
- CutWeeds()
  : Farm


- Generated on Thu Jan 10 2013 13:15:39 for ALMaSS Skylark ODdox by
   1.8.1.1
